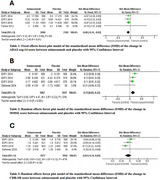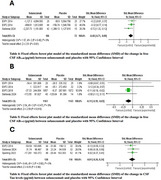# Efficacy of Solanezumab in Early Alzheimer’s Disease: A Systematic Review and Meta Analysis

**DOI:** 10.1002/alz70859_103207

**Published:** 2025-12-25

**Authors:** Youssef M. Seddeik, Muataz El‐Barbari, Mohamed Nasim, Mahmoud Orhan, Youssef A. Ismail

**Affiliations:** ^1^ Faculty of Medicine Port Said Univeristy, Egypt, Port Said, Port Said Egypt

## Abstract

**Background:**

Alzheimer's disease (AD) is a neurodegenerative disease and one of the main causes of dementia posing a significant global burden. The anti‐Aβ monoclonal antibody (mAb) solanezumab was used in the treatment of early AD in recent trials and have produced mixed cognitive and clinical results. The purpose of this systematic review and meta‐analysis is to investigate the effect of solanezumab on cognitive functions and biomarkers levels in RCTs on AD patients.

**Methods:**

A systematic search was conducted up to December 2024 across databases such as PubMed, Scopus, Web of Science, Cochrane library and ClinicalTrials.gov for RCTs. The quality of studies was assessed using the Cochrane risk of bias 2 tool. RevMan 5.3 was used for statistical analysis. Cognitive functions included in meta‐analysis were MMSE, ADAS‐Cog14, and CDR‐SB. Biomarkers included in meta‐analysis were CSF free AB1‐40, CSF free AB1‐42, and CSF Tau levels.

**Results:**

Six randomized clinical trials with a total of 5472 enrolled participants were included. All Biomarkers levels demonstrated negative outcomes for the solanezumab group compared to the placebo group; CSF free AB1‐40, CSF free AB1‐42 and CSF Tau showed standardized mean difference of ‐0.40[‐0.71, ‐.0.09], ‐0.11[‐0.19, ‐0.03] and ‐0.01[‐0.26, 0.24] respectively, indicating that solanezumab does not provide clinically significant results. Solanezumab mostly showed statistically significant improvement, although limited, across cognitive tests including MMSE and ADAS‐Cog14 ‐0.08[‐0.14, ‐0.02] and 0.13 [0.05, 0.22] respectively, while CDR‐SB test didn’t favor either groups ‐0.04[‐0.14, 0.05].

**Conclusion:**

Despite solanezumab worsening biomarkers levels, the data suggests that solanezumab has a limited efficacy to improve the cognitive function of participants with early AD.